# Rapamycin and fasting sustain autophagy response activated by ischemia/reperfusion injury and promote retinal ganglion cell survival

**DOI:** 10.1038/s41419-018-1044-5

**Published:** 2018-09-24

**Authors:** Rossella Russo, Giuseppe Pasquale Varano, Annagrazia Adornetto, Francesca Nazio, Gianluca Tettamanti, Rossana Girardello, Valentina Cianfanelli, Federica Cavaliere, Luigi Antonio Morrone, Maria Tiziana Corasaniti, Francesco Cecconi, Giacinto Bagetta, Carlo Nucci

**Affiliations:** 1 Department of Pharmacy, Health and Nutritional Sciences, Section of Preclinical and Translational Pharmacology, University of Calabria, 87036 Arcavacata di Rende, Italy; 2 Ophtalmology Unit, Department of Experimental Medicine, University of Rome “Tor Vergata”, 00133 Rome, Italy; 3 Department of Pediatric Hematology and Oncology, IRCSS Bambino Gesù Children’s Hospital, Rome, Italy; 4 Department of Biotechnology and Life Sciences, University of Insubria, 21100 Varese, Italy; 5 Cell Stress and Survival Unit, Center for Autophagy, Recycling and Disease (CARD), Danish Cancer Society Research Center, 2100 Copenhagen, Denmark; 6 Department of Health Sciences, University “Magna Graecia” of Catanzaro, 88100 Catanzaro, Italy

## Abstract

Autophagy, the cellular process responsible for degradation and recycling of cytoplasmic components through the autophagosomal–lysosomal pathway, is fundamental for neuronal homeostasis and its deregulation has been identified as a hallmark of neurodegeneration. Retinal hypoxic–ischemic events occur in several sight-treating disorders, such as central retinal artery occlusion, diabetic retinopathy, and glaucoma, leading to degeneration and loss of retinal ganglion cells. Here we analyzed the autophagic response in the retinas of mice subjected to ischemia induced by transient elevation of intraocular pressure, reporting a biphasic and reperfusion time-dependent modulation of the process. Ischemic insult triggered in the retina an acute induction of autophagy that lasted during the first hours of reperfusion. This early upregulation of the autophagic flux limited RGC death, as demonstrated by the increased neuronal loss observed in mice with genetic impairment of basal autophagy owing to heterozygous ablation of the autophagy-positive modulator *Ambra1* (*Ambra1*^*+/gt*^*)*. Upregulation of autophagy was exhausted 24 h after the ischemic event and reduced autophagosomal turnover was associated with build up of the autophagic substrate SQSTM-1/p62, decreased ATG12-ATG5 conjugate, ATG4 and BECN1/Beclin1 expression. Animal fasting or subchronic systemic treatment with rapamycin sustained and prolonged autophagy activation and improved RGC survival, providing proof of principle for autophagy induction as a potential therapeutic strategy in retinal neurodegenerative conditions associated with hypoxic/ischemic stresses.

## Introduction

Autophagy is a highly conserved catabolic process responsible for degradation of cytoplasmic content^1^. During macroautophagy (hereafter referred to as autophagy), cytosolic components are sequestered in a double-membrane vesicle called autophagosome and delivered to lysosomes^2,3^. The pathway regulates the physiological turnover of long-lived proteins and organelles, and acts as a quality control mechanism by clearing protein aggregates and dysfunctional organelles^4^. Furthermore, through the mobilization of intracellular resources, autophagy enables cells to adapt to stressful environments, allowing the survival under states of increased metabolic requirement or reduced nutrient availability^5^. On the other hand, autophagy overactivation, and the consequent self-digestion, has been associated with cellular death^6^.

Owing to their post-mitotic nature, high energy demand and distinctive morphology, neurons are strictly dependent on autophagy efficiency^7^, and several studies have shown that maintaining the appropriate level of autophagy is fundamental for neuronal health^8–10^. Indeed, alterations of autophagy recur in a variety of neurodegenerative pathologies^11–13^. Retinal ganglion cells (RGCs) are the primary output neurons of vertebrate retina and their loss occurs in several eye diseases, including retinopathy of prematurity^14,15^, diabetic retinopathy^16,17^, central retinal artery occlusion^18^, and glaucoma^19^, with the last being the second cause of irreversible blindness worldwide^20^. Hypoxic/ischemic events are common in several of the above disorders^21,22^ and retinal hypoperfusion has been shown to occur in glaucoma patients, this contributing to the initiation and progression of the neuropathy^23,24^.

In our previous work, we described the calpain-mediated cleavage of the autophagy related protein BECN1/beclin1 in rat retina following an ischemic insult and suggested a deregulation of autophagy under this experimental conditions^25^. However, the mechanisms and the role of the observed modulation still remain controversial. Indeed, we and others reported in vitro and in vivo evidence for a neuroprotective role of autophagy in retinal neurons^26–28^, whereas other groups have shown opposite results^29,30^.

Here, with the aim to validate the hypothesis of inducing autophagy as a potential strategy to achieve neuroprotection, we first depicted the time-window and molecular mechanisms of autophagy modulation in the ischemic retina and then used the acquired information to design the most appropriate approach to modulate the pathway.

## Materials and methods

### Animals

Male C57BL/6 J mice (25–30 g) were purchased from Charles River (Lecco, Italy), male *Ambra1*^*+/gt*^^31^ and GFP-LC3 mice^32^ were provided by Professor Cecconi (Rome, Italy), and housed with a 12 h light–dark cycle with ad libitum access to food and water. Animals assigned to the fasting protocol were deprived of food with free access to water for 24 or 48 h; body weight was monitored at the beginning and at the end of the fasted period. Animal care and experimental procedures were carried out in accordance to the guidelines of the Italian Ministry of Health (D.L. 26/2014), the European Communities Council Directive (2010/63/UE) and the ARVO Statement for the Use of Animals in Ophthalmic and Vision Research. The experimental protocol was approved by the Italian Ministry of Health (Rome; NIH license no. 1026/2016-PR). All surgical procedures were performed under deep anesthesia and efforts were made to minimize the number of animals used and their suffering.

### Cells

Murine embryonic fibroblast (MEFs) primary cells were prepared from E13.5 embryos, cultured in Dulbecco’s modified Eagle’s medium (DMEM, Sigma-Aldrich, Milan, Italy) supplemented with 20% fetal calf serum (FCS, Sigma-Aldrich, Milan, Italy), 2 mM l-glutamine, 1% penicillin/streptomycin solution at 37 °C under 5% CO_2_. Cells were utilized for experiments at the second passage in culture. *Ambra1* expression levels correspond to half dosage in *Ambra1*^*+/gt*^ cells, as well as in the corresponding animals, if compared with wild-type cells/animals^33^

### Retinal ischemia

Retinal ischemia was induced in the right eye (I, ischemic) by acute increase of the intraocular pressure (IOP) according to the method previously reported^21,34–36^. Animals were deeply anesthetized by intraperitoneal injection of Xilazin (Rompun®, Bayer Spa, Milan, Italy), Tiletamin-Zolazepam (Zoletil®, Virbac Srl, Milan, Italy) mixture and laid on a heating pad to maintain the body temperature at 37 °C. Topical anesthesia was induced by 0.4% Oxibuprocain eye drops (Novesina®, Novartis Farma, VA, Italy). A 29-gauge infusion needle, connected to a 500 ml bottle of sterile saline, was inserted in the anterior chamber of the right eye, and the saline container was elevated to produce an intraocular pressure of 90–100 mmHg for 60 min. For each animal, left eye (C, control) was used as non-ischemic control.

Body temperature was monitored and animals with values lower than 35.5 °C were excluded from the study. Mice were killed at 0, 1, 6, 24 h, or 7 days of reperfusion. To minimize the basal variations due to the circadian autophagy regulation^37^, animals were all killed between 1.00 and 3.00 pm. For western blot analysis both eyes were immediately enucleated and retinas quickly dissected, snap frozen in liquid nitrogen, and stored at − 80 °C until use.

### Drug administration

Rapamycin (cat. nr. R5000; LC Laboratories, Woburn, MA, USA) was dissolved in 100% ethanol and stored at − 20 °C. Rapamycin (10 mg/Kg; Zhou et al., 2009) or vehicle (10% ethanol, 5% PEG400, and 5% Tween 80) were injected intraperitoneally once a day for 6 consecutive days. Retinal ischemia was induced on the fifth day of treatment and animals were killed after 24 h or 7 days of reperfusion.

### Autophagic flux

Retinas were rapidly isolated at the indicated time points, chopped with a vannas scissors and placed in a 24-well plate with RPMI-1640 medium (Gibco, Life Technologies, Paysley, UK) in presence or absence of ammonium chloride (NH_4_Cl, 20 mM) and leupeptin (Leu, 200 μM; Sigma-Aldrich, Milan, Italy) as lysosomal enzymes activity inhibitors^3,38^. NH_4_Cl and leupeptin stock solution were prepared in water at the concentration of 2 M and 10 mM, respectively. Samples were incubated at 37 °C in humidified atmosphere of 5% CO_2_ for 2 h. Tissue suspension was centrifuged at 5000×*g* for 5 min at 4 °C; the supernatant was discarded and the tissue homogenized with a pestle (Sigma-Aldrich, Milan, Italy) in 25 μl of ice-cold RIPA buffer containing protease (cod. P8349; Sigma-Aldrich, Milan, Italy) and phosphatase (cod. 524625, Calbiochem, La Jolla, CA, USA) inhibitor cocktails. The homogenate was centrifuged for 15 min, 10,000 g at 4 °C, and the supernatant assayed for protein content by the Bio-Rad DC Protein Assay Kit (Bio-Rad Laboratories, Milan, Italy) and subjected to immunoblot analysis.

For the autophagy flux assay in MEFs, cells were cultured in DMEM supplemented with 20% FCS, or in Earle’s balanced salt solution (EBSS), in presence or absence of the lysosomal inhibitor chloroquine (Clq; Sigma-Aldrich, Milan, Italy)^3^. Clq stock solution was prepared in water at the concentration of 20 mM and cells were treated with 20 μM Clq for 30 min, in DMEM supplemented with 20% FCS, or in EBSS. To analyze GFP-LC3 dots, cells grown on coverslips were fixed with 4% paraformaldehyde in phosphate-buffered saline (PBS), washed three times and then examined under a Delta Vision Fluorescent Microscope (Olympus). The results indicate the number of GFP-LC3-positive cells (cells with more than 10 GFP-LC3 punctate dots). 80 cells per sample were counted.

### Protein extraction and western blotting

Retinas were lysed in ice-cold RIPA buffer (50 mM Tris-HCl (pH 8), 150 mM NaCl, 1 mM thylenediaminetetraacetic acid, 0.1% sodium dodecyl sulfate, 1% IGEPAL, 0.5% Sodium deoxycholate) containing protease (cod. P8349; Sigma-Aldrich, Milan, Italy) and phosphatase (cod. 524625, Calbiochem, La Jolla, CA, USA) inhibitor cocktails. Lysates were centrifuged for 15 min, 10,000 g at 4 °C, and supernatants assayed for protein content by the Bio-Rad DC Protein Assay Kit (Bio-Rad Laboratories, Milan, Italy).

Equal amount of total proteins were separated by sodium dodecyl sulfate–polyacrylamide gel electrophoresis, transferred onto PVDF membranes (Immobilon-P, Sigma-Aldrich, Milan, Italy) and blocked with 5% non-fat milk or 5% BSA (bovine serum albumin, Sigma-Aldrich) in Tris-buffered saline containing 0.05% Tween 20 for 1 h at room temperature. Primary antibodies were incubated overnight at 4 °C followed by a species-specific horseradish peroxidase conjugated goat IgG as secondary antibody (Pierce Biotechnology, Rockford, IL, USA) for 1 h at room temperature. A list of the primary antibodies used is reported in Table 1. Protein bands were visualized with Western Blotting Luminol Reagent (Santa Cruz Biotechnology, Dallas, USA) and the chemiluminescence signal detected using X-ray films (Santa Cruz Biotechnology, Dallas, USA). Autoradiographic films were scanned, digitalized at 600 dpi, and band quantification was performed using ImageJ software (NIH, Bethesda, MD, USA).

**Table 1:** Sources and dilutions of primary antibodies

Target	Supplier and catalog no.	Method and dilution
LC3	MBL, PM036	WB, 1:2000
p62	Sigma, P0067	WB, 1:4000
p62	Santa Cruz, sc-25575	IFC, 1:50
Beclin1	Cell Signaling, 3495	WB, 1:1000
4EBP1	Cell Signaling, 9644	WB, 1:1000
p-4EBP1 (Thr37/46)	Cell Signaling, 2855	WB, 1:1000
ULK1	Cell Signaling, 8054	WB, 1:1000
p-ULK1 (Ser757)	Cell Signaling, 6888	WB, 1:1000
mTOR	Cell Signaling, 2972	WB, 1:1000
p-mTOR (Ser2448)	Cell Signaling, 2971	WB, 1:1000
AMPK	Cell Signaling, 2603	WB, 1:1000
pAMPK (Thr172)	Cell Signaling, 2535	WB, 1:1000
ATG4B	Cell Signaling, 5299	WB, 1:1000
ATG7	Cell Signaling, 2631	WB, 1:1000
ATG12	Cell Signaling, 4180	WB, 1:1000
β-actin	Sigma, A5441	WB, 1:30000
GAPDH	Applied Biosystem, AM4300	WB, 1:30000
LAMP-2	Pierce Biotechnology, PA1-655	IFC, 1:50
TUJ1	BioLegend, MMS-435P	IFC, 1:500

*WB* western blotting, *IFC* immunofluorescence

### Immunofluorescence

After induction of retinal ischemia, mice were killed at the indicated time points. Eyes were enucleated and fixed in 4% paraformaldehyde at 4 °C for 1 h, cryopreserved in 15% sucrose overnight and then in 30% sucrose for 1 week^39^. Specimens were frozen in Optimal Cutting Temperature compound (Tissue-Tek®, Sakura Finetek Europe, The Netherlands), and 14 μm cryostat sections were cut, mounted onto Superfrost Plus glass slides (Thermo Fisher Scientific, Waltham, MA, USA) and stored − 80 °C until use.

For detection of GFP-LC3 signal, sections were washed in 0.1 M PBS (pH 7.4) and mounted with Vectashield mounting media with 4′,6-diamidino-2-phenylindole (DAPI) (Vector Laboratories, Burlingame, CA, USA). For cellular and subcellular localization of specific antigens, retinal sections were thawed, air-dried, post-fixed in 4% paraformaldehyde for 15 min and washed in 0.1 M PBS (pH 7.4). Sections were permeabilized with 0.3% Triton-X100 (Sigma-Aldrich, Milan, Italy) for 1 h and blocked with 10% donkey serum (Sigma-Aldrich, Milan, Italy) at room temperature for 1 h. Slides were incubated with primary antibody in 5% donkey serum overnight (primary antibodies used are listed in Table 1) followed by incubation with anti-rabbit Alexa Fluor 555, 1:500 (Molecular Probes, Eugene, OR, USA) at room temperature for 1 h and mounted with Vectashield mounting media with DAPI (Vector Laboratories, Burlingame, CA, USA). Image acquisition was performed using a confocal microscope (Leica TC-SP2 Confocal System; Leica Microsystems, Milan, Italy).

### Transmission electron microscopy

Mice were killed 6 or 24 h following the ischemic injury and eyes enucleated. Retinas were fixed in Karnowsky fixative (4% paraformaldehyde and 2.5% glutaraldehyde in 0.1 M phosphate buffer, pH 7.4) for 4 h at 4 °C, and then post-fixed with 1% osmium tetroxide in 0.1 M phosphate buffer for 1 h at room temperature. After standard ethanol dehydration, samples were embedded in an Epon-Araldite 812 mixture. Ultrathin sections (80-nm-thick) were obtained with a Reichert Ultracut S ultratome (Leica, Nussolch, Germany). After staining with uranyl acetate and lead citrate, samples were observed with a JEM-1010 transmission electron microscope (Jeol, Tokyo, Japan). TEM images were acquired with a Morada digital camera.

### Retrograde labeling of RGCs

To evaluate cell loss, RGCs were retrogradely labeled by stereotaxically injecting the fluorescent tracer FluoroGold (FG; Fluka, Sigma-Aldrich, Milan, Italy) into the superior colliculi^40^. Four days after the ischemic insult, mice were deeply anaesthetized, immobilized in a stereotaxic device (Kopf 900, Analytical Control, Milan, Italy) and the positions of superior colliculi were identified using the Paxinos and Watson atlas (1998). The skull was exposed and 2 µl of 2% FluoroGold solution were injected on both sides of the skull 4 mm posterior to the bregma, 1 mm lateral to the sagittal suture and 1.6 mm ventral from the bone surface using a Hamilton Neuros-syringe with a 33-gauge needle (Hamilton Europe, Bonaduz, Switzerland). The skin was then sutured and a 0.3% tobramycin ointment was applied (Tobral®, Alcon, Milan, Italy). Animals were killed 7 days after ischemia and eyeballs enucleated and fixed for 20 min in paraformaldehyde 4% in PBS. The timing of FG injection (after injury) and the time elapsed between the dye application and the processing of the retina were chosen based on previous studies; this experimental setting also prevents the labeling of activated microglia^41,42^. The anterior segment of the eye was removed and the posterior eye-cup additionally fixed for 30 min. Isolated retinas were divided into four quadrants (superior, inferior, nasal and temporal) and mounted on slides using the ProLong Gold Antifade Mountant (Thermo Fisher Scientific, Waltham, MA, USA). Thirty-two images per retina (three from the peripheral, three from the middle, and two from the central retina for each quadrant) were acquired using a deconvolution microscope (Leica Microsystems CMS EL6000, GBH, Mannheim, Germany) at × 40 magnification and subjected to cell count using ImageJ software (NIH, Bethesda, MD, USA) by blind investigators. The total number of labeled cells in the ischemic eye was compared with contralateral eye and expressed as percentage of RGC loss.

### Statistical analysis

Data were expressed as mean ± standard error of the indicated number of independent experiments and evaluated statistically for difference by analysis of variance followed by Tukey–Kramer test for multiple comparisons. Where indicated, Student’s *t* test was used to evaluate differences between two means. A value of *P* < 0.05 was considered significant.

## Results

### Retinal ischemia induces a reperfusion time-dependent modulation of autophagic flux

Following conjugation with phosphatydilethanolamine, cytosolic protein LC3I (microtubule-associated protein light chain 3 I) is converted into LC3II that stably associates with the autophagosomal membrane^43^.

**Fig. 1:**
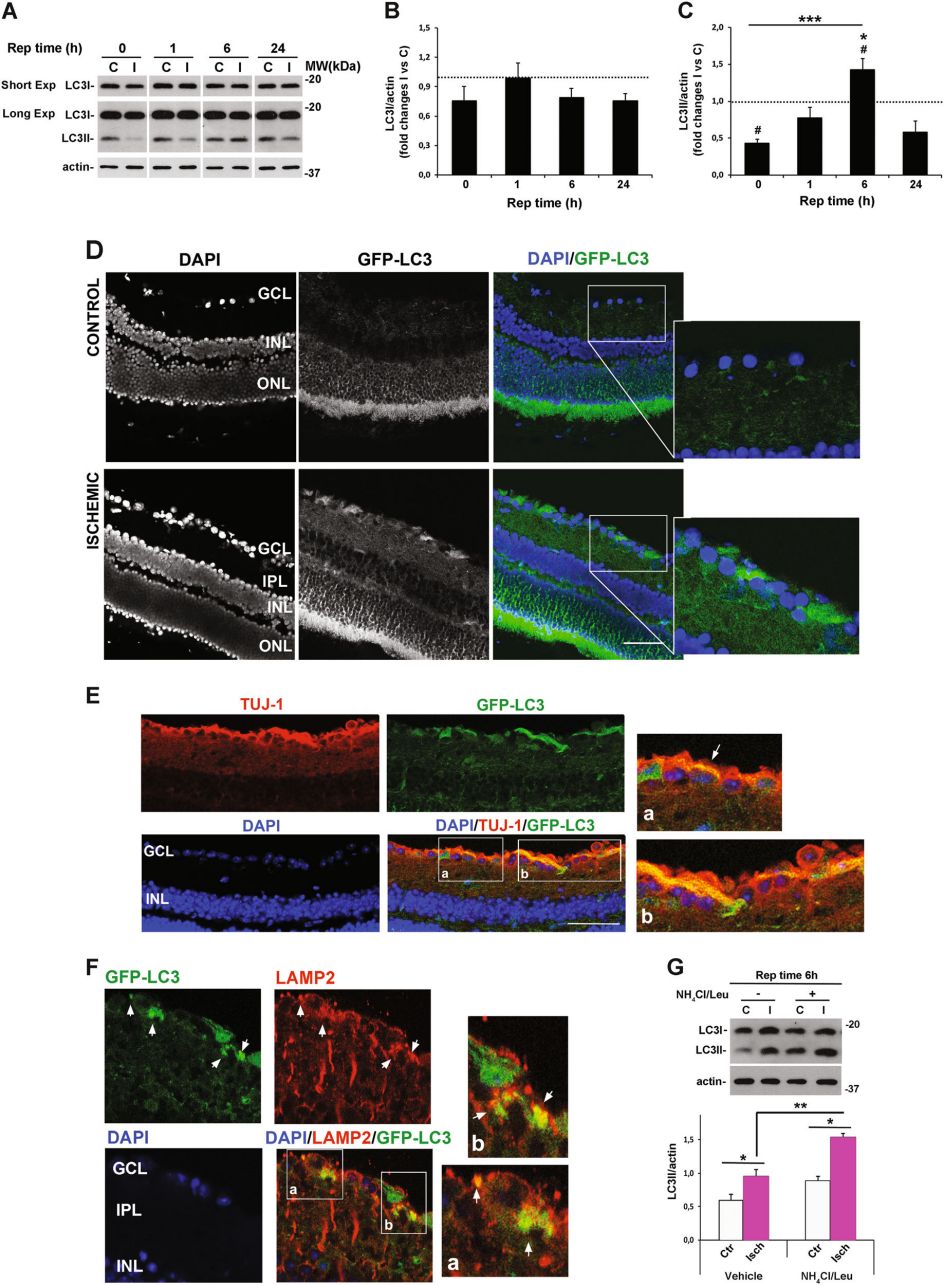
Modulation and distribution of LC3 following retinal ischemia/reperfusion injury. Mice were subjected to retinal ischemia in the right eye (I) for 60 min and killed after 0, 1, 6, or 24 h. For each animal, retina from contralateral eye was used as control. **A** Immunoblot showing the time-dependent modulation of LC3 expression in whole retinal lysates at the indicated time of reperfusion (Rep time). Histograms represent the densitometric analysis of the bands expressed as **B** LC3I and **C** LC3II normalized to loading control (actin). Dashed lines indicate the baseline expression of the protein in non-ischemic retinas set to 1. Data are reported as mean ± s.e.m. (4–6 independent experiments for each group). ^#^*P* < 0.05 vs control non-ischemic retina (Student’s *t* test); *P < 0.05 vs 1 and 24 h of reperfusion (ANOVA followed by Tukey–Kramer multiple comparisons test); ***P < 0.001 vs 0 h of reperfusion (Student’s *t* test). C, control non-ischemic retina; I, ischemic retina; MW, molecular weight; Short Exp, short exposure; Long Exp, longer exposure. **D** Confocal images showing the upregulation of endogenous fluorescence in retinas of GFP-LC3 transgenic mice subjected to ischemia and killed after 6 h of reperfusion. The inserts are higher magnification photomicrographs showing the signal distribution in GCL and IPL. **E** colocalization of GFP-LC3 signal with the RGC marker TUJ1 (red) in ischemic retinas reperfused for 6 h. **F** Representative retinal tissue sections from GFP-LC3 transgenic mice showing the partial colocalization of lysosomal marker LAMP-2 (red) with GFP-LC3-positive round-shaped vesicles (white arrowhead) at the ganglion cell layer (GCL) of the ischemic retina. Images are representative of three animals per experimental conditions. Frozen tissue sections were prepared as described in the methods and nuclei counterstained with DAPI (blue). GCL, ganglion cell layer; IPL, inner plexiform layer; INL, inner nuclear layer; ONL, outer nuclear layer. Scale bars **D** 50 μm, **E** 47.62 μm **F** 50 μm. **G** Immunoblot showing the ex vivo analysis of autophagic flux in retinas subjected to ischemia (I, Isch) followed by 6 h of reperfusion as compared with non-ischemic retinas (C, Ctr). Samples from individual retinas were split in half and incubated for 2 h in medium with (NH_4_Cl/Leu) or without (vehicle) ammonium chloride (NH_4_Cl, 20 mM) and leupeptin (Leu, 200 μM) to inhibit lysosomal enzymatic activity. Histograms show the densitometric analysis of the bands normalized on internal control (actin). Data are reported as mean ± s.e.m. of three independent experiments. *P < 0.05, **P < 0.01 (Student’s *t* test)

Ischemia and the following reperfusion have opposite effects on LC3II levels, whereas LC3I is not significantly affected (Fig. 1A, B). The ischemic insult applied to the right eye (I, ischemic; reperfusion time 0) induced a significant reduction of LC3II (Fig. 1A–C) as compared with the left non-ischemic retina (C, control). During the first hour of reperfusion, LC3II recovered toward basal value (set to 1 in the figure) and further accumulated at 6 h (Fig. 1A–C).

In GFP-LC3 mice subjected to retinal ischemia followed by 6 h of reperfusion, increased endogenous fluorescence was mostly evident in the inner retinal layers (ganglion cell layer, GCL and inner plexiform layer) (Fig. 1D). The enrichment in GFP-LC3-positive round-shaped structures was marked in RGCs as demonstrated by the colocalization of the endogenous fluorescence with the RGC marker TUJ1 (Fig. 1E).

Immunofluorescence for lysosomal-associated membrane protein 2 (LAMP-2) showed that, in the ischemic retina, a fraction of GFP-LC3 accumulated in LAMP-2-positive vesicles, suggesting that autophagolysosomal structures are formed in the ischemic retina (Fig. 1F).

To distinguish if the observed LC3II increase was due to enhanced autophagosomal formation or reduced autophagosomal turnover we performed ex vivo autophagic flux (Fig. 1G). Control and ischemic retinas from mice subjected to 6 or 24 h reperfusion were incubated with or without NH_4_Cl and leupeptin, two lysosomal inhibitors^3^. When lysosomal activity was inhibited, a significant increase of LC3II was reported in both control and ischemic retinas subjected to 6 h reperfusion, as compared with vehicle (Fig. 1G). However, the extent of LC3II accumulation was significantly higher in the ischemic retinas as compared with contralateral, suggesting that autophagosome clearance was upregulated in the retina isolated at this time point. On the contrary, no LC3II increase was reported upon lysosomal inhibition in retinas subjected to 24 h reperfusion (data not shown).

p62/SQSTM-1 (sequestrosome 1) is an autophagy receptor that links ubiquitinated proteins/organelles to LC3 and it is loaded with the autophagic cargo into the autophagosomes. Therefore, increased autophagic flux leads, in general, to p62 depletion, whereas the protein accumulates when autophagic degradation is inhibited^3,44,45^.

**Fig. 2:**
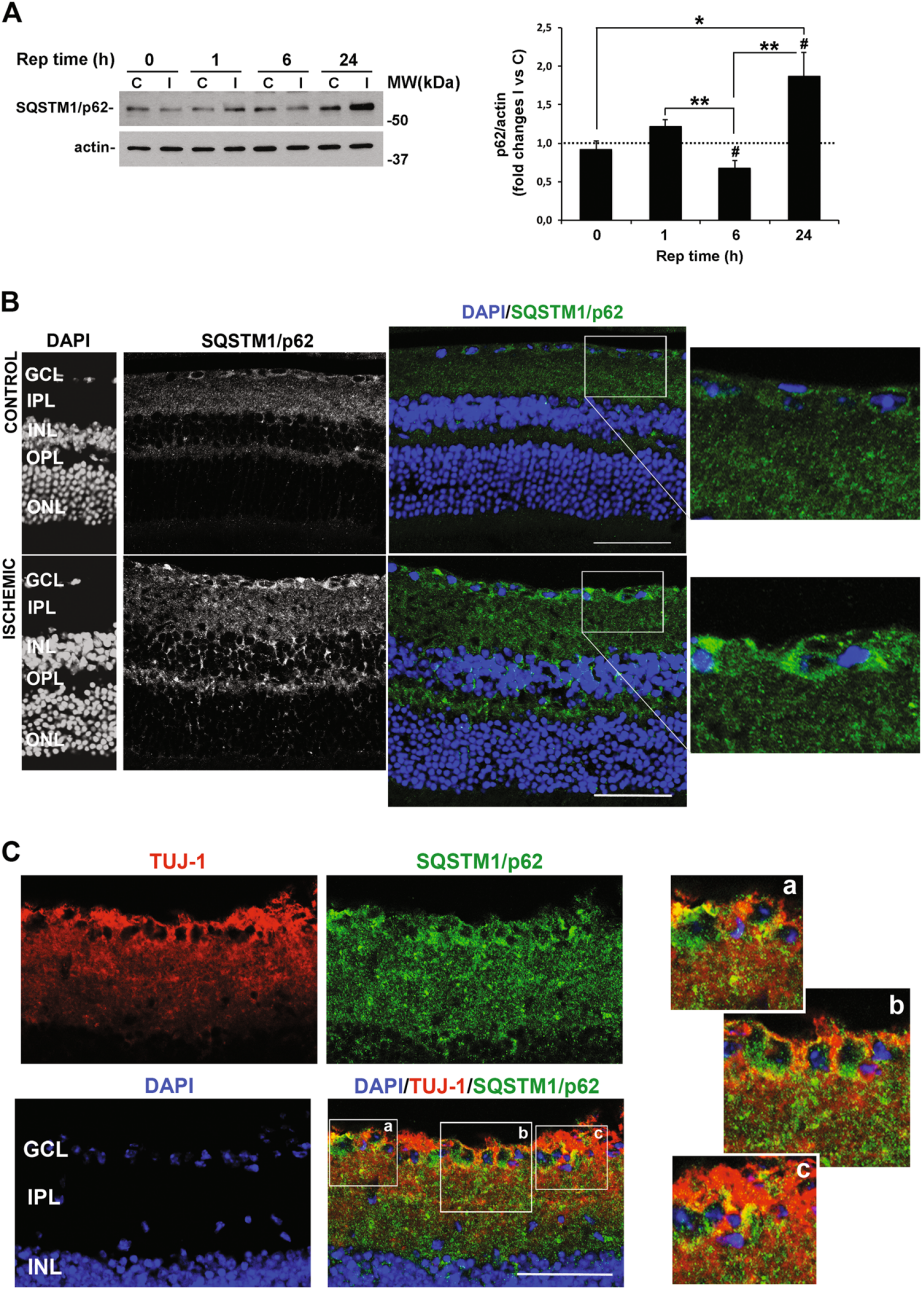
Changes of SQSTM-1/p62 levels following retinal ischemia reperfusion. **A** Western blotting analysis reporting the reperfusion time-dependent modulation of SQSTM-1/p62. SQSTM-1/p62 expression decreased in the ischemic retina as compared with contralateral after 6 h of reperfusion, whereas accumulated at 24 h. Histograms show the densitometric analysis of the bands normalized to loading control (actin) and reported as mean ± s.e.m. (3–6 independent experiments for each group). Dashed line indicates the baseline expression of the protein of interest in control non-ischemic retinas set to 1. ^#^*P* < 0.05 vs C (Student’s *t* test); *P < 0.05, **P < 0.01 (ANOVA followed by Tukey–Kramer for multiple comparisons test). **C**, control non-ischemic retina; I, ischemic retina; MW, molecular weight; Rep time, reperfusion time. **B** Representative retinal tissue sections showing SQSTM-1/p62 immunoreactivity in control and ischemic retinas after 24 h of reperfusion. **C** Colabeling of ischemic retina at 24 h reperfusion with anti-p62 (green) and anti-TUJ1 (red), a RGC-specific marker, demonstrating p62 upregulation in RGC soma (GCL) and dendrites (IPL). Nuclei were counterstained with DAPI (blue). Images are representative of three animals per experimental conditions ONL, outer nuclear layer; OPL, outer plexiform layer; INL, inner nuclear layer; IPL, inner plexiform layer; GCL, ganglion cell layer. Scale bars **B** 50 μm; **C** 47.62 μm

In our experimental conditions, upregulation of LC3II at 6 h of reperfusion was associated with a significant reduction of p62/SQSTM-1, a result consistent with autophagy induction (Fig. 2A). Conversely, following 24 h reperfusion, we reported a pronounced increase of p62/SQSTM-1 in retina homogenates (Fig. 2A). At this time point, p62/SQSTM-1 immunoreactivity was upregulated in the innermost layers where it partially colocalized with TUJ1-positive RGCs (Fig. 2B, C).

### Ultrastructural features of autophagy in retinas subjected to ischemia/reperfusion injury

**Fig. 3:**
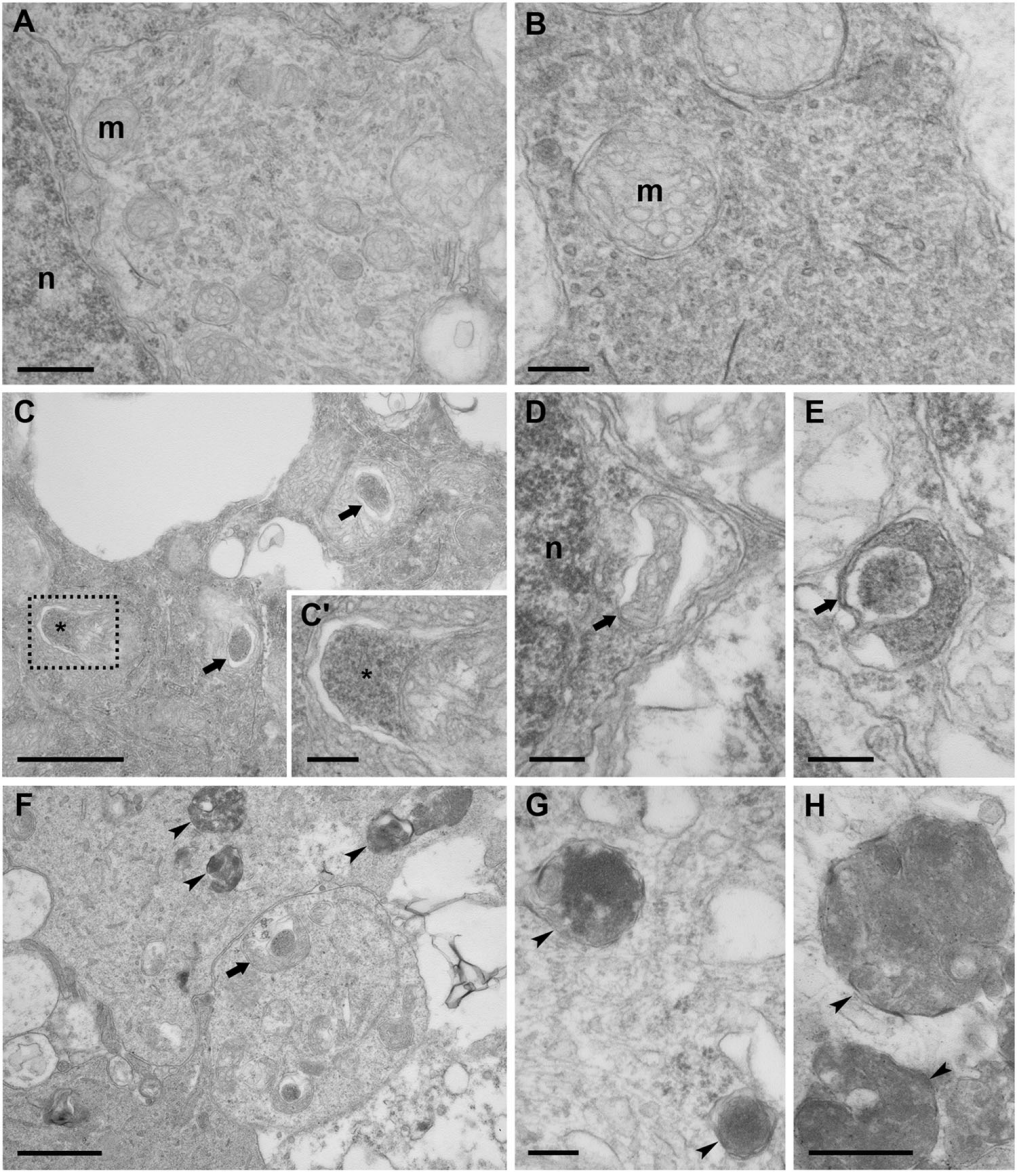
Ultrastructural analysis of retinas after ischemia/reperfusion injury. **A**, **B** TEM micrographs showing the cytoplasm of cells in GLC. **C–E** In retinas subjected to 6 h reperfusion, abundant autophagosomes (arrows), characterized by a double-limiting membrane, are detected in the cells. **F–H** After 24 h reperfusion, autophagic compartments (arrowheads) accumulating in the cytoplasm are visible. Asterisk: phagophore; m: mitochondria; n: nucleus. Boxed area in **c** is shown at higher magnification in **c**’. Scale bars 500 nm **A**, **H**; 200 nm **B**, **D**, **C**’, **E**, **G**; 1 μm **C**, **F**

The observation of double-membrane compartments by transmission electron microscopy represents the gold standard for identifying autophagosomes^3^. Although cells in the GLC from non-ischemic control showed a normal cytoplasm, devoid of vacuoles, in which mitochondria were easily recognizable (Fig. 3A, B), in the ischemic retina subjected to 6 h reperfusion, numerous double-membrane vacuoles were observed **(**Fig. 3C–E**)**. In addition to mature autophagosomes containing electron-dense material (Fig. 3D, E), phagophores, involved in the initial step of autophagosome formation, could be detected (Fig. 3C, C’). In retina subjected to 24 h reperfusion, accumulation of autophagic compartments was observed (Fig. 3F–H) corroborating the hypothesis that the build up of p62 (see Fig. 2C) in the GCL was due to reduced autophagy efficiency.

### Changes of upstream ATG proteins following retinal ischemia/reperfusion

**Fig. 4:**
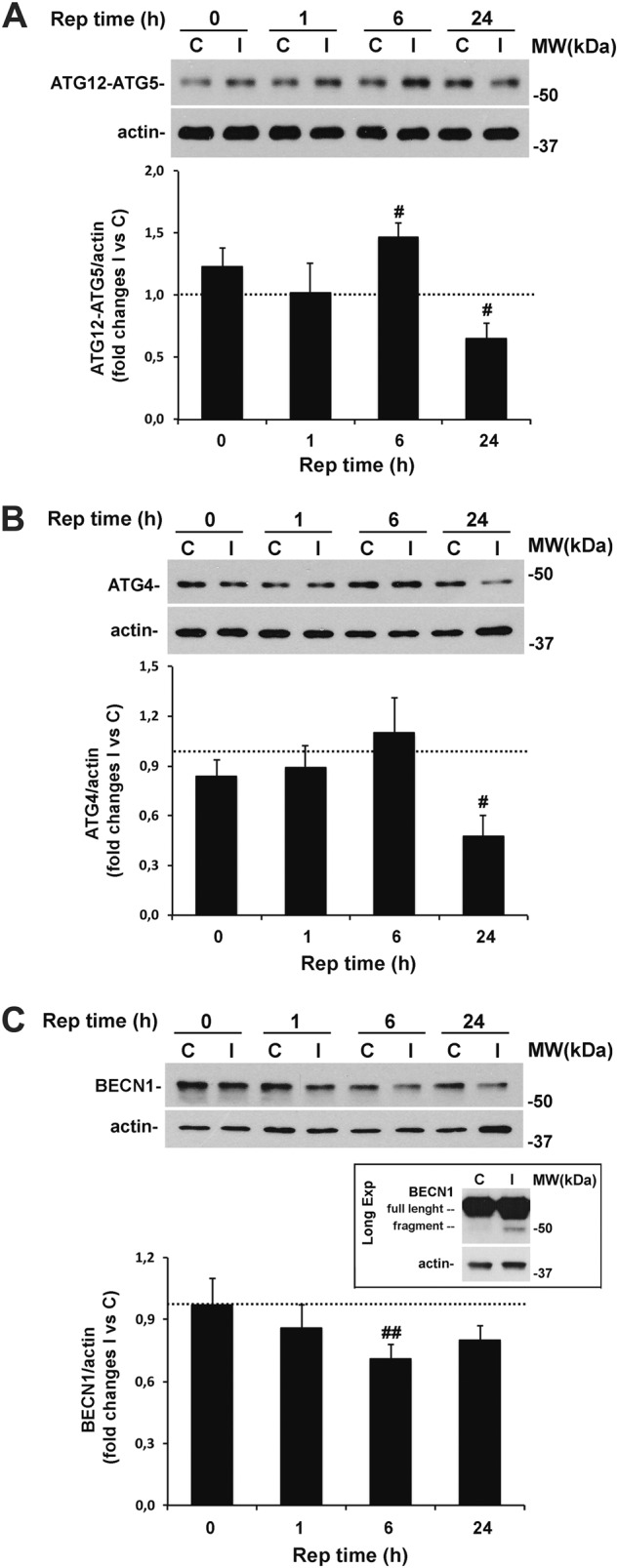
Time-dependent changes of ATG proteins expression following ischemia/reperfusion injury. Immunoblotting of **A** ATG12-ATG5 conjugate and **B** ATG4 showing a significant decrease of the indicated proteins in the ischemic retina after 24 h of reperfusion. **C** Reduced levels of BECN1 were reported in the retinas subjected to ischemia plus 6 h of reperfusion and associated with the accumulation of a 50 kDa proteolytic fragment (figure box). Note that fragment was detectable only by longer exposure time leading to the saturation of the full-length band (long exp, longer exposure). For each animal, retina from contralateral eye was used as control. Histograms represent the densitometric analysis of the bands normalized to loading control (actin). Dashed lines indicate the baseline expression of the protein in non-ischemic retinas set to 1. Data are reported as mean ± s.e.m. (3–4 independent experiments for each group). ^#^*P* < 0.05, ^##^*P* < 0.01 vs. control non-ischemic retina (Student’s *t* test). **C**, control non-ischemic retina; I, ischemic eye; MW, molecular weight; Rep time, reperfusion time

Covalent conjugation of ATG12 to ATG5 mediates the vesicle expansion and promotes LC3 lipidation^46^, whereas ATG4 is involved in the processing and lipidation of LC3^47^. A significant increase of ATG12-ATG5 conjugates was observed in the ischemic retinas following 6 h of reperfusion, whereas a significant reduction was detected at 24 h as compared with contralateral non-ischemic retina (Fig. 4A). Similarly, ATG4 expression dropped in the ischemic retina after 24 h of reperfusion (Fig. 4B).

The BECN1 (ATG6)/class III phosphoinositide 3-kinase (Vsp34) complex participates to the membrane nucleation step preceding the autophagic vesicle formation^48^. We recently showed that, in retinas of rats subjected to ischemia, BECN1 is reduced during the post-ischemic phase owing to calpain-mediated proteolytic cleavage^25^. Similarly, here we observed a significant time-dependent decrease of BECN1 that was significant at 6 h of reperfusion and accompanied by the appearance of the proteolytic fragment (Fig. 4C).

### Autophagy modulation by retinal ischemia/reperfusion is associated with changes in the activation state of mTOR and AMPK pathways

Autophagy activity inversely correlates with the activation state of the mammalian target of rapamycin (mTOR) and the mTOR complex 1 (mTORC1) formation that, when active, phospho-inhibits Unc51-like kinase 1 (ULK1) complex preventing autophagy^49,50^. To verify the possibility of targeting mTOR to achieve retinal autophagy modulation, we monitored the time-dependent changes of mTOR signaling between 0 and 24 h of reperfusion.

**Fig. 5:**
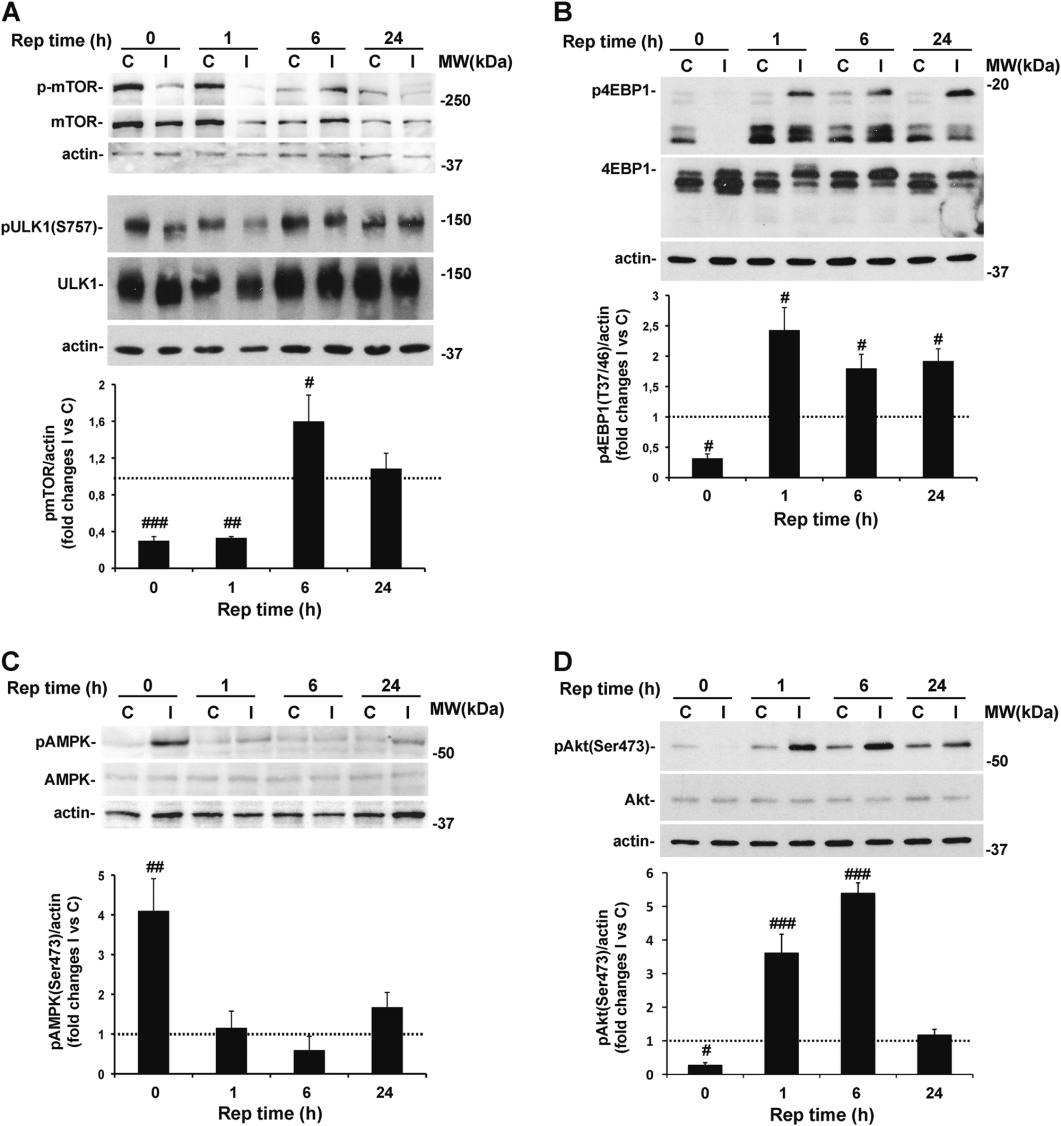
Time-dependent modulation of mTOR signaling pathway upon retinal ischemia. Retinal ischemia was induced in the right eye and mice were killed after 0, 1, 6, or 24 h of reperfusion. For each animal, contralateral non-ischemic retina was used as control. The phosphorylation level of **A** mTOR (p-mTOR), **C** AMPK (pAMPK), and **D** Akt (p-Akt) was studied in whole retinal lysates by western blotting. mTOR activity was indirectly checked by analyzing the state of phosphorylation of its downstream targets **A** ULK1 and **B** 4EBP1. Histograms represent the densitometric analysis of the bands normalized to loading control (actin). Dashed lines indicate the baseline expression of the protein in non-ischemic retinas set to 1. Data are reported as mean ± s.e.m. of 3–7 independent experiments for each group. ^#^*P* < 0.05, ^##^*P* < 0.01, ^###^*P* < 0.001 vs control non-ischemic retina (Student’s *t* test). **C**, control non-ischemic retina; I, ischemic retina; MW, molecular weight; Rep time, reperfusion time

As shown in Fig. 5, retinal ischemia induced a transient dephosphorylation of mTOR (Ser^2448^), which corresponded with the kinase deactivation, as confirmed by the decreased phosphorylation of two mTOR downstream targets, ULK1 (Ser^757^) and 4EBP1 (Thr^37/46^) (Fig. 5A, B). The opposite effect was detected at 6 h of reperfusion, when a significant upregulation of phospho-mTOR was evident as compared with contralateral non-ischemic retina (Fig. 5A). At this time point, activation of mTOR was associated with the return to basal levels of p-ULK and increased p-4EBP1 (Fig. 5A, B). The latter was maintained through the following 24 h of reperfusion (Fig. 5B).

Opposite to mTOR, the serine/threonine AMP activated kinase (AMPK), which is activated under low-energy conditions, promotes autophagy through inhibition of mTORC1 and activation of ULK1^51–53^. AMPK phosphorylation (Thr^172^) was significantly upregulated in the injured retina as compared with contralateral (Fig. 5C) and returned to basal level within 1 h of reperfusion (Fig. 5C).

mTOR is a downstream target of the phosphatidylinositol 3-kinase (PI3K)/Akt pathway^54^. Once phosphorylated on Ser^347^, Akt directly phosphorylates mTOR on Ser2448, activates mTORC1 and inhibits autophagy^55^. Under our experimental setting, retinal ischemia induced a transient dephosphorylation of Akt (Fig. 5D), followed by a significant increase of p-Akt that peaked at 6 h; after 24 h, p-Akt levels in the ischemic retina were comparable to contralateral non-ischemic tissue (Fig. 5D).

### Systemic administration of rapamycin activates retinal autophagy and decreases RGC loss induced by ischemia

**Fig. 6:**
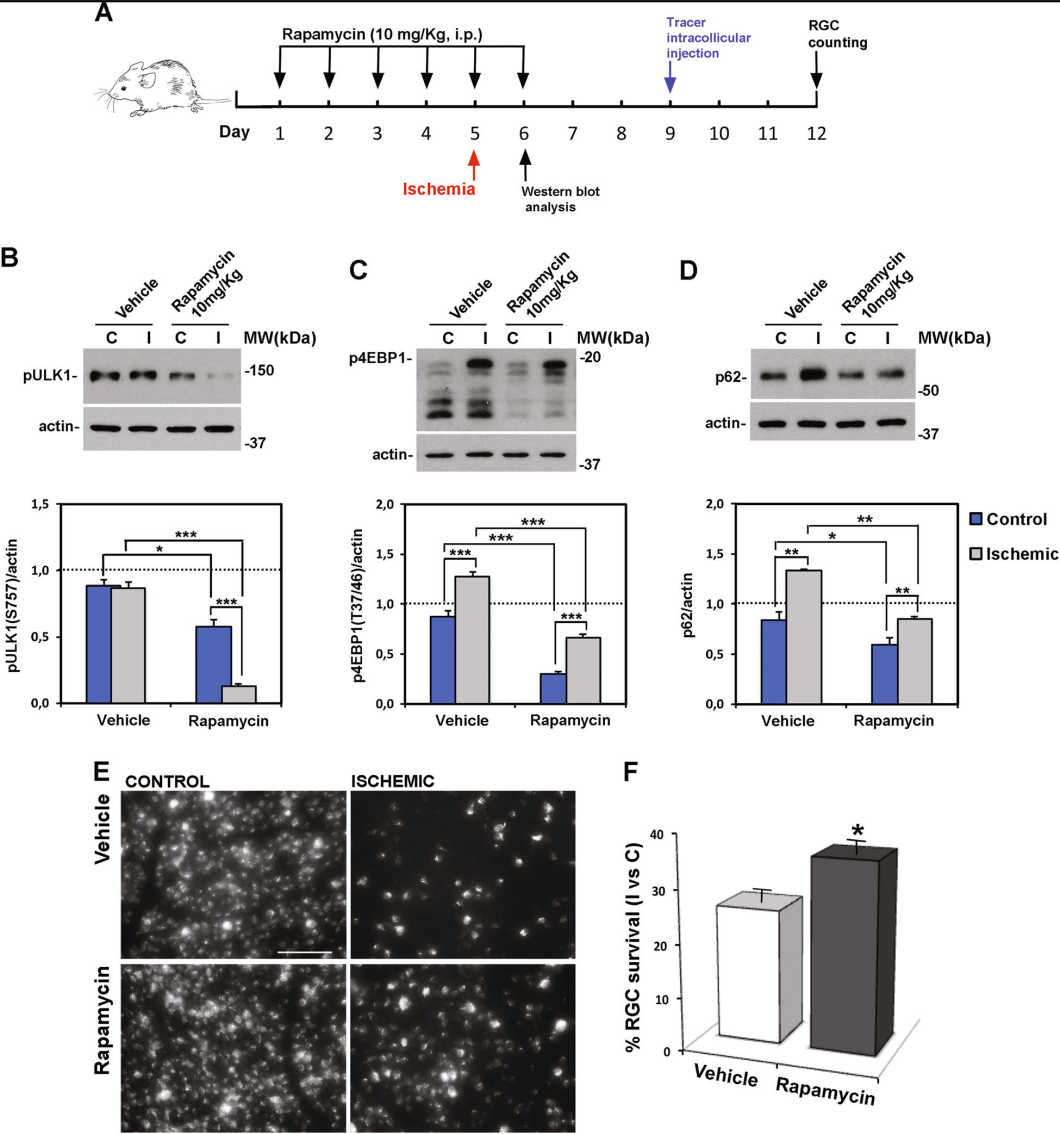
Rapamycin promotes autophagy and increases RGC survival following retinal ischemia/reperfusion. **A** Rapamycin treatment schedule. Rapamycin (10 mg/Kg) or vehicle were injected i.p. once a day for 6 consecutive days; ischemia was induced the fifth day and mice killed after 24 h for the biochemical analysis **B**, **C**, **D** or 7 days for evaluation of RGC survival **E**, **F**. Western blotting analysis of **B** ULK and **C** 4EBP1 phosphorylation levels was performed to indirectly check the inhibition of mTOR activity in the retina of rapamycin-treated mice. p-ULK (S757) and p4EBP (T37/46) were reduced in control and ischemic retinas from rapamycin-treated mice as compared with both control and ischemic retinas of vehicle-treated mice. **D** Rapamycin reduced basal p62 expression in non-ischemic retina as compared with vehicle-treated control and prevented the accumulation of p62 in the ischemic retinas at 24 h of reperfusion. Histograms represent the densitometric analysis of the bands normalized by the internal loading control (actin). Data are reported as mean ± s.e.m. of three independent experiments for each group. *P < 0.05, **P < 0.01, ***P < 0.001 (ANOVA followed by Tukey–Kramer for multiple comparisons test). **C**, control non-ischemic retina; I, ischemic retina; MW, molecular weight. **E** Representative fluorescent photomicrographs of whole-mount ischemic and control retinas from vehicle and rapamycin-treated mice. Systemic treatment with rapamycin significantly increased the percentage of FluoroGold-labeled RGCs in the ischemic retinas as compared with vehicle-treated animals. Images are representative of three independent experiments. Scale bar 75 μm. **F** Histogram reports the result of RGC count. Twenty images per retina were acquired and the total number of labeled cells in the ischemic retina **I** was compared with contralateral, non-ischemic retina **C** and expressed as percentage of RGC survival. Results are reported as mean ± s.e.m. of three independent experiments. *P < 0.05 (Student’s *t* test)

Based on the modulation of mTOR pathway following retinal ischemia/reperfusion injury, we used the mTOR inhibitor rapamycin to pharmacologically enhance autophagy in the retina. Mice were treated with rapamycin for 6 consecutive days, starting 5 days before the induction of ischemia (Fig. 6D). To ascertain that, under the described posology, effective dose of rapamycin reached the retina, phosphorylation of mTOR substrates was checked. As reported in Fig. 6, p-ULK1 (Ser^757^) and p-4EBP1 (Thr^37/46^) were reduced in the control retinas of animal treated with rapamycin, confirming the effective inhibition of mTOR (Fig. 6B, C). The reduced level of p62/SQSTM-1 in the left retinas of treated mice further substantiated the efficacy of the treatment and the induction of autophagy (Fig. 6D).

Accordingly, in the ischemic retinas, treatment with rapamycin significantly reduced the upregulation of p-4EBP1, maintained lower levels of p-ULK1 ^Ser757^, and reduced the accumulation of p62/SQSTM-1 observed 24 h after the insult (Fig. 6B–D). We also reported that rapamycin administration increased Akt phosphorylation (figure S2).

These biochemical changes were associated with a significant increase of FluoroGold-labeled RGCs in the ischemic retina of rapamycin-treated mice as compared with vehicle-treated (Fig. 6E, F), thus supporting the neuroprotective role of autophagy.

### Effect of fasting on ATG proteins expression in the retina

**Fig. 7:**
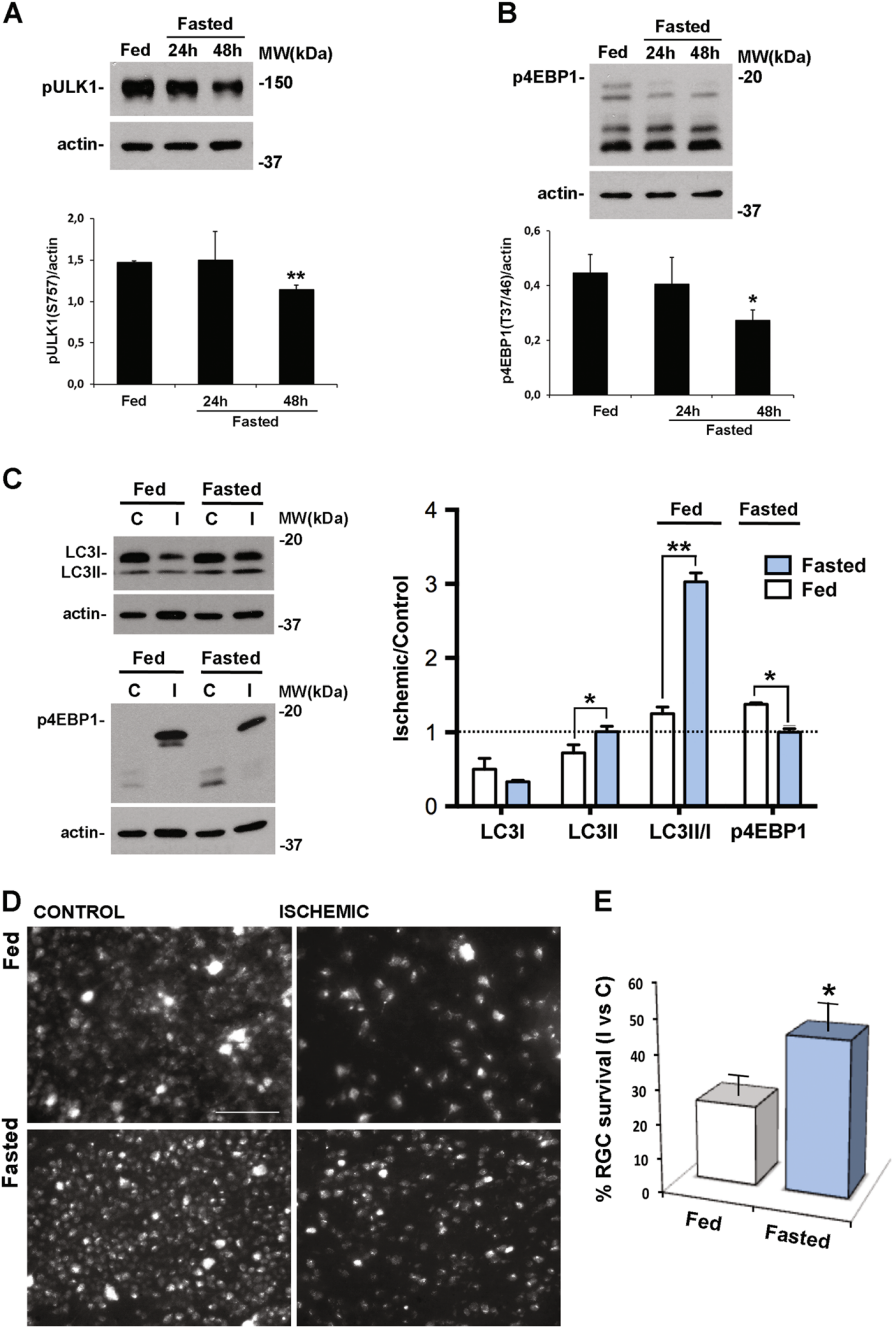
Fasting downregulates mTOR activity and upregulates autophagy in the retina and prevents RGC loss induced by ischemia/reperfusion injury. Representative western blotting showing the downregulation of **A** ULK1 and **B** 4EBP1 phosphorylation in naive retina from mice subjected to 48, but not 24, hours fasting as compared with retinas from fed animals. In **C**, **D**, and **E** animals fasted for 48 h were subjected to retinal ischemia and killed after 24 h **C** or 7 days **D**, **E**. **C** Western blot analysis showing a significant increase of LC3II in both control and ischemic retinas from fasted animals as compared with fed. Significant decrease of the phosphorylated form of 4EBP1 (p-4EBP1) in the ischemic retinas of fasted mice as compared with fed was also reported. The results of the densitometric analysis of the autoradiographic bands reported in the graph show the comparison between the relative levels of the protein of interest in fasted vs fed animals. Values were normalized to loading control (actin). Data are shown as mean ± s.e.m. of 3–4 independent experiments for each experimental group. *P < 0.05, **P < 0.01 vs Fed (Student’s *t* test). c, control eye; I, ischemic eye; MW, molecular weight **D** Representative fluorescent photomicrographs of whole-mount ischemic and control retinas from fasted and fed animals. Fasting significantly increased the percentage of FluoroGold-labeled RGCs in the ischemic retinas as compared with fed animals. Images are representative of three independent experiments. Histogram in **E** reports the quantification of RGC survival under the different diet regimens. Thirty-two images per retina were acquired and the total number of labeled cells in the ischemic retina **I** was compared with contralateral, non-ischemic retina **C**, and expressed as percentage of RGC survival. Results are reported as mean ± s.e.m. of three independent experiments. *P < 0.05 (Student’s *t* test). Scale bar 75 μm

To further support the evidence of the neuroprotective role of autophagy, we attempt to induce retinal autophagy by fasting. Since in eukaryotic cells nutrient starvation induces autophagy through inhibition of mTOR signaling^56^, to prove the effectiveness of fasting in the retina we looked for changes of p-ULK1 (Ser^757^) in naive mice food-restricted for 24 or 48 h. A significant reduction of p-ULK1 was detectable in the 48 h fasted mice, whereas no significant changes were reported in mice fasted for 24 h (Fig. 7A). Deactivation of mTOR was further confirmed by a significant reduction of p-4EBP1 (Thr^37/46^) following 48, but not 24 h fasting (Fig. 7B).

Analysis of ATG proteins in retinas of mice fasted for 48 h showed a significant upregulation of ATG12/ATG5 conjugate, while no significant changes were reported for BECN1, ATG4, and ATG7 (figure S1).

In the retina of mice subjected to ischemia, 48 h fasting reduced the phosphorylation of 4EBP1 (Thr^37/46^) observed after 24 h reperfusion (Figs. 7C and 5B) suggesting that, at this time point, a low mTOR activation state was maintained in the ischemic retina of fasted mice as compared with fed (Fig. 7C). As shown in Fig. 7C, LC3II was upregulated in both control and ischemic retinas from food-restricted mice. Furthermore, in the ischemic retina allowed to reperfuse for 24 h, upregulation of LC3II was significantly higher in fasted mice as compared with mice with free access to food (fed) (Fig. 7C).

### Fasting prevents RGC death following ischemia/reperfusion injury

To determine the effect of fasting on RGC loss extent, retinal ischemia was induced in mice subjected to food restriction. Seven days after the insult a significant increase of surviving RGCs was reported in the retina of mice fasted for 48 h as compared with normal fed (45.1 ± 8.7% vs 23.4 ± 5.7%) (Fig. 7D, E); the neuroprotective effect was absent in mice food-restricted for 24 h (data not shown).

### Partial autophagic impairment by heterozygous genetic ablation of Ambra1 increases RGC loss induced by retinal ischemia

**Fig. 8:**
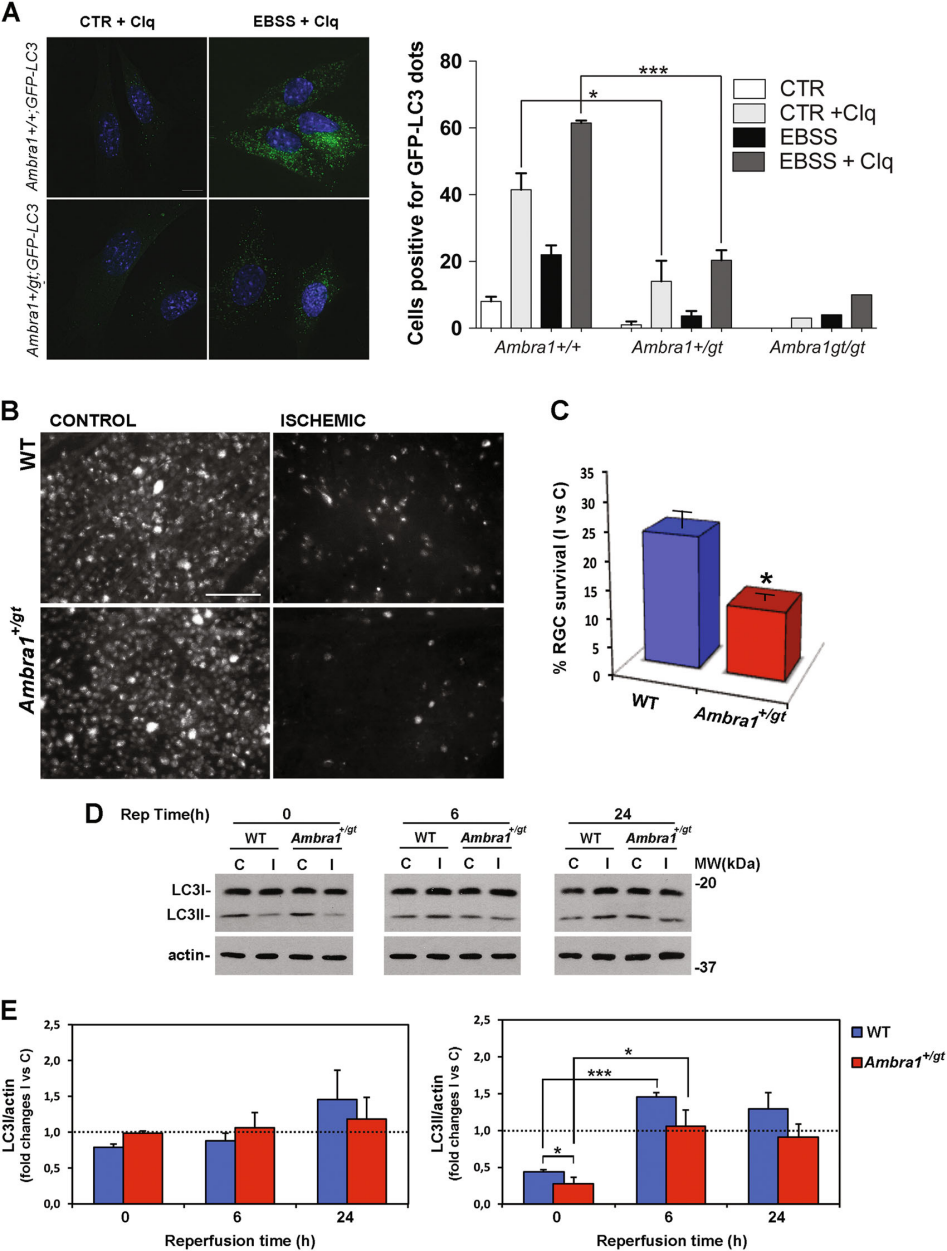
Reduced basal autophagy in Ambra1^+/gt^ mice increases RGC death induced by ischemia/reperfusion injury. **A** murine embryonic fibroblasts (MEFs) dissected from Ambra1^+/+^; GFP-LC3 (*n* = 2), Ambra1^+/gt^; GFP-LC3 (*n* = 3) and Ambra1^gt/gt^; GFP-LC3 (*n* = 1) embryos were grown in control (CTR) or starvation medium (EBSS, for 30 min). Where indicated, 20 μM chloroquine was added to CTR/EBSS media. The number of cells positive for GFP-LC3 dots is reported in the graph (cells with more than 10 dots were considered positive for GFP-LC3 dots). Scale bar: 10 µM. Bars represent mean ± s.e.m. with 80 cells analyzed per sample. *P < 0.05; ***P < 0.0005 (Student’s *t* test). **B** Representative fluorescent photomicrograph of whole-mount retinas showing the reduction of FluoroGold-labeled RGCs in the ischemic retina of autophagy deficient Ambra1^+/gt^ mice as compared with non-ischemic contralateral retina and ischemic retinas from wild-type mice (WT). Scale bar 75 μm. Histograms in **C** report the quantification (expressed as % of survival) of RGC 7 days after the injury in the ischemic retina **I** as compared with contralateral non-ischemic retina **C**. RGC survival was significantly decreased in autophagy deficient mice (Ambra1^+/gt^) as compared with WT. Results are reported as mean ± s.e.m. of three independent experiments. *P < 0.05 (Student’s *t* test). **D** Representative immunoblotting showing changes of LC3 expression in Ambra1^+/gt^ transgenic mice subjected to retinal ischemia as compared with wild type. Animals were killed at 0, 6, or 24 h of reperfusion. **D** Histograms represent the densitometric analysis of the bands normalized to loading control (actin). Dashed lines indicate the baseline expression of the protein in non-ischemic retinas set to 1. Data are reported as mean ± s.e.m. (4–6 independent experiments for each group). *P < 0.05, **P < 0.01, ***P < 0.001 (ANOVA followed by Tukey–Kramer for multiple comparisons test). **C**, control non-ischemic eye; I, ischemic eye; MW, molecular weight; Rep time, reperfusion time

Autophagy/beclin1 regulator 1 (AMBRA1) acts as a positive regulator of autophagy by facilitating BECN1/VPS34 interaction and stabilizing ULK1^57,58^. AMBRA1 is a very upstream regulator of autophagy, being inhibited mTOR and activated by ULK1^58^. Also, its deficiency has a strong phenotype on the developing nervous system, despite its ubiquitous expression^31^. In order to assess whether the heterozygous genetic disruption of *Ambra1* is sufficient to impair autophagy, we crossed mice heterozygous (*Ambra1*^*+/gt*^) for the gene-trap mutation in the *Ambra1* locus with mice expressing the autophagy reporter GFP-LC3, and analyzed the autophagy flux in primary MEFs isolated from the resulting embryos (wild type - *Ambra1*^*+/+*^*;GFP-LC3* - or heterozygous - *Ambra1*^*+/gt*^*;GFP-LC3* - for *Ambra1*). As reported in Figure 8 A, autophagy flux is impaired in *Ambra1*^*+/gt*^*;GFP-LC3* with respect to *Ambra1*^*+/+*^*;GFP-LC3* cells, as demonstrated by the lower number of GFP-LC3 dots observed in *Ambra1* heterozygous *vs*. wild-type cells, upon chloroquine treatment. Also, we found that *Ambra1* dosage affects both basal (control) and starvation-induced (EBSS) autophagy (Fig. 8A). Given its key role in autophagy upstream regulation, its dose-dependent impact on autophagy and its key importance for neuronal growth control and survival^31^, we decided to investigate whether a deficiency of autophagy could affect RGC survival. To this aim, retinal ischemia was induced in *Ambra1*^*+/gt*^ and the number of FluoroGold-labeled RGC evaluated. As reported in Fig. 8C, the value of RGC survival in the ischemic retina of wild-type mice 7 days after the insult was 28.0 ± 3.3% as compared with the fellow control retina. Heterozygous ablation of *Ambra1* resulted in a significant reduction of RGC survival following ischemia (12.0 ± 1.2%) (Fig. 8B, C), suggesting that partial genetic impairment of basal autophagy depletes RGCs from a relevant endogenous neuroprotective mechanism.

At the biochemical level, retinas from *Ambra1*^*+/gt*^ mice showed a more pronounced decrease of LC3II at the end of the ischemia (reperfusion time 0), and a reduced recovery of LC3II in the following 6 h (Fig. 8C, D).

## Discussion

Retinal hypoxic–ischemic events occur in several sight-treating disorders such as central retinal artery occlusion, diabetic retinopathy, and glaucoma^22^, leading to RGC death and visual impairment. By using a mouse model of retinal ischemia, here we showed that ischemic insult triggers an acute autophagy response lasting the initial hours of reperfusion. This induction contains RGC death, as demonstrated by the increased RGC loss observed in mice genetically unable to implement this response. Upregulation of autophagy is exhausted 24 h after ischemia and its prolonged activation, by rapamycin or fasting, sustained RGC survival, thus providing proof of principle for autophagy induction as a therapeutic strategy for retinal neurodegenerative conditions involving hypoxic/ischemic stresses.

The recycling of cellular components through autophagy is essential for cellular homeostasis and it represents a cytoprotective mechanism allowing cells to cope with stressing conditions^59^. In our study, a significant decrease of the autophagosome-associated form of LC3 (LC3II)^43^ was observed at the end of the ischemic period; as LC3II is degraded with the cargo content, this data might reflect a robust induction of autophagy triggered by ischemia^60,61^. The subsequent increase of LC3II, together with the decrease of the autophagic substrate p62/SQSTM-1 and the accumulation of GFP-LC3 puncta, pointed to the occurrence of an increased autophagic flux. In this phase of reperfusion the increased incidence of autophagosomal structures in the cytoplasm of cells located in the GCL and positive for RGC markers, suggest that autophagic flux is enhanced in this cell type. In view of autophagy as mechanism of self-adaptation to cellular stresses, this initial autophagy response might represent the attempt to preserve cellular homeostasis and limit the damage. Indeed, when this reaction was impaired, as in mice with autophagy deficiency (*Ambra1*^*gt/+*^*)*^31^, the extent of RGC death increased. Consistently, in a mouse model of optic nerve transection, RGC survival was reduced in ATG4B knockout mice or following specific deletion of ATG5 in RGCs^26^.

Upregulation of LC3II and accumulation of autophagosomes have been reported in RGCs following exposure to glaucoma-related stimuli, although the time path varies depending on the initial insult^12^. Increased LC3 immunoreactivity has been reported in GCL between 6 and 24 h after ischemia in rats^29,30^. In particular, Wei and colleagues showed a persistent upregulation of LC3 and increased number of autophagosomal structures in RGCs lasting until 7 days post injury^30^. Conversely, here we showed that, following 24 h of reperfusion, accumulation of LC3II is no longer detectable in the ischemic retina and comparable levels of the protein accumulate in control and ischemic retinas in the presence of lysosomal activity inhibitors, this suggesting that autophagy induction is eventually exhausted. In support of this, we observed a decrease of ATG proteins (i.e., ATG4, ATG12-5, BECN1) involved in earlier steps of autophagy and a build up of p62/SQSTM-1 in the cytoplasm of RGCs. Decline of autophagy turnover was further confirmed by the recurrence in RGC of autophagic compartments containing partially degraded cytoplasmic material and mitochondria.

Impairment of the autophagic flux with accumulation of p62/SQSTM-1 has been reported in RGC axons following IOP elevation by laser photocoagulation in rats^62^. Similarly, we observed accumulation of p62/SQSTM-1-positive bodies in RGCs, suggesting a defect in protein clearance by autophagy. However, as p62/SQSTM-1 also targets ubiquitin-modified proteins to the proteasome^63^ and it is subjected to transcriptional regulation^64^ the contribution of these processes in the reported accumulation cannot be ruled out.

Macroautophagy and mitophagy are negatively regulated by the mTORC1^50^, mainly via inhibition of the ULK1 complex^65^.In our experimental setting, autophagy induction coincided with inactivation of mTOR and its upstream modulator Akt^55^. Concomitantly, we observed a transient activation of AMPK, a cellular energy sensor that inhibits mTOR^51^ and activates ULK1^53^. These data suggest that inhibition of mTOR, by either inactivation of the PI3K/Akt pathway and activation of AMPK, is involved in the mechanisms of autophagy induction triggered by retinal ischemia.

During reperfusion, AMPK is no longer active, whereas a sustained phosphorylation of Akt can be observed. Therefore, the maintenance of the upregulated autophagy flux observed in the early phase of reperfusion takes place in the absence of mTOR inhibition and might rely on different molecular events or representing the final part of the autophagy wave triggered by ischemia. Furthermore, the transient overactivation of mTOR observed after 6 h of reperfusion, might act as a brake on autophagy activation, slowing down the cargo clearance^66^. The efficiency of the autophagic pathway, as the time of reperfusion progresses, might be further compromised by BECN1 cleavage^25^.

Currently there are not unified views regarding the role of autophagy in RGC death; likewise, pharmacological treatments modulating autophagy in animal models of RGC degeneration led to controversial results^12^. Here we showed that rapamycin, whereas prolonged autophagic flux induction, also attenuated RGC loss, thus supporting the neuroprotective role of autophagy. Consistently, rapamycin reduced loss of RGCs after optic nerve transection^26^, improved RGC survival following chronic ocular hypertension and reduced apoptosis in glutamate-injured primary RGCs^28^. On the contrary, rapamycin exacerbated RGC death following optic nerve ischemia^67^ and, in adult RGCs, conditional deletion of PTEN, a negative regulator of mTOR pathway, increased survival and promoted axon regeneration after optic nerve injury^68^. These opposite outcomes might reside on the different posology other than the type of detrimental stimulus applied. Furthermore, owing to the multifunctional role of mTOR, rapamycin might affect other intracellular pathways. For example, the rapamycin-mediated increased phosphorylation of Akt, a pro-survival factor that is relevant for RGC survival^69,70^, might take part to the observed neuroprotection.

However, the significant reduction of RGC loss observed in fasted mice strengthens the neuroprotective role of autophagy in the retina exposed to hypoxic/ischemic insults. Caloric restriction is the most physiological trigger of autophagy and the *dogma* of a neuronal resistance to starvation-induced autophagy^32,71^ has been recently refuted by evidence reporting autophagy upregulation in the brain and retina of food-restricted mice^39,72–76^. Here we showed that 48 h of food deprivation activated autophagy in the retina; a shorter fasting period (24 h) was ineffective in inducing autophagy and failed in affording neuroprotection to RGCs (data not shown). These results differ from what has been reported by Esteban-Martinez and colleagues^39^, who showed activation of autophagy in all retinal layers of mice food-restricted for 24 h. One possible explanation for the different results may arise from the fact that this study was conducted in naive retinas and in a different mouse strain (wild type vs GFP-LC3).

The neuroprotective effect of fasting reported here has two main implications: (1) it supports the hypothesis that inducing autophagy in insulted retina has neuroprotective effects and (2) suggests that short-term food restriction might represent a potential intervention for the treatment of retinal neurodegenerative disorders, in particular those, like glaucoma, where current available therapies are not sufficient to halt the disease. This is also supported by a recent study showing that 7 weeks of every other day fasting suppresses retinal degeneration in a mouse model of normal tension glaucoma^77^. Moreover, a retrospective cohort study showed that the risk of developing open-angle glaucoma was reduced in diabetic patients taking the antidiabetic metformin, a caloric restriction mimetic drug that has been shown to induce autophagy in several systems^78^.

Altogether, our data add knowledge to the autophagy dynamic in the retina under hypoxic/ischemic conditions, define autophagy as a determinant for RGC survival and identify this pathway as an important endogenous neuroprotective mechanism that can be targeted for neuroprotection.

## Electronic supplementary material

Supplemental 1

Supplemental 2

Supplementary materials
