# Supplementary figures and images for: Rapamycin and fasting sustain autophagy response activated by ischemia/reperfusion injury and promote retinal ganglion cell survival
Source: Cell Death Dis. 2018 Sep 24;9(10):981. doi: 10.1038/s41419-018-1044-5 (PMC6155349; doi:10.1038/s41419-018-1044-5)

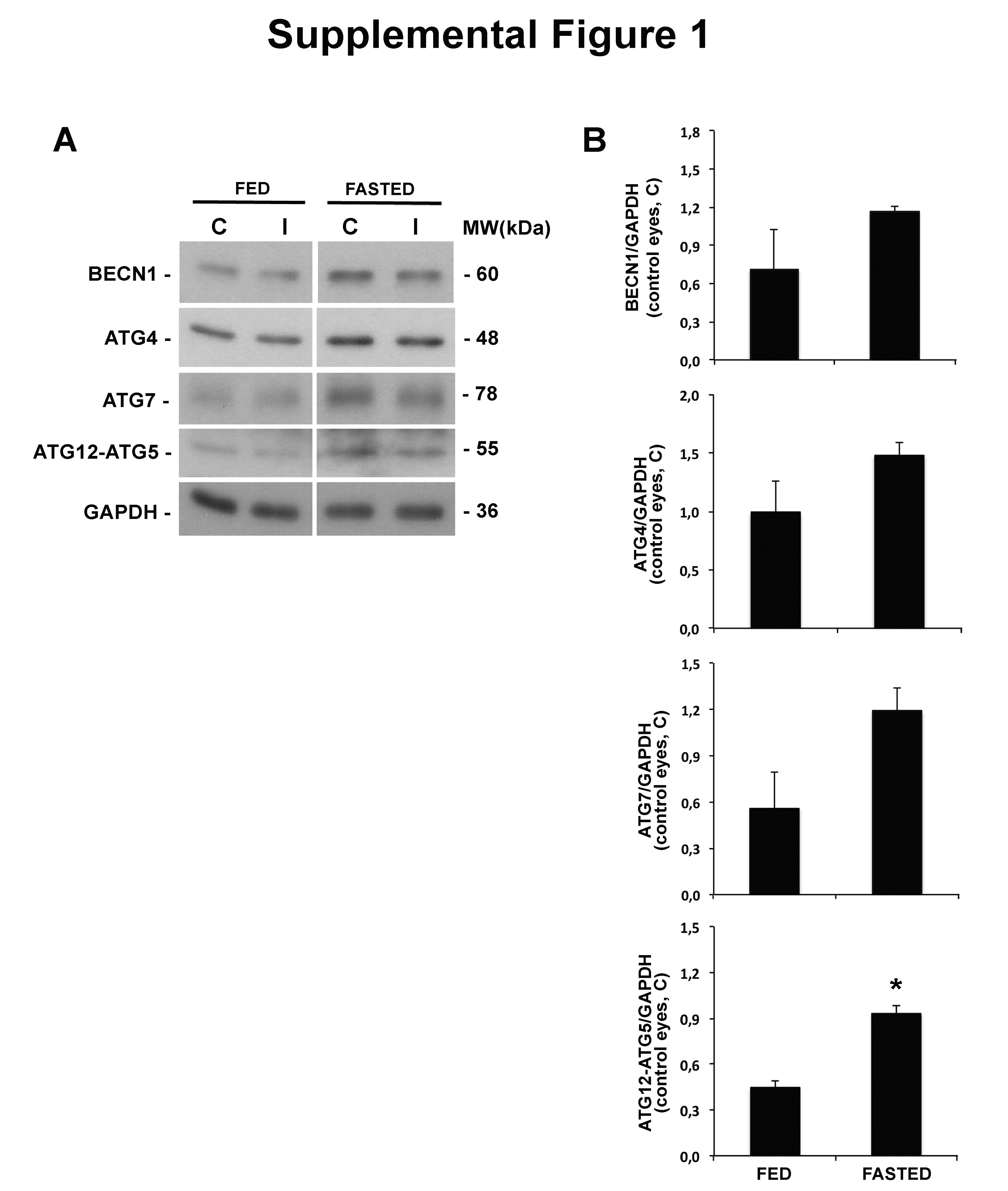

Supplement: Supplementary file 1 — Supplemental 1 [file 41419_2018_1044_MOESM1_ESM.tif]

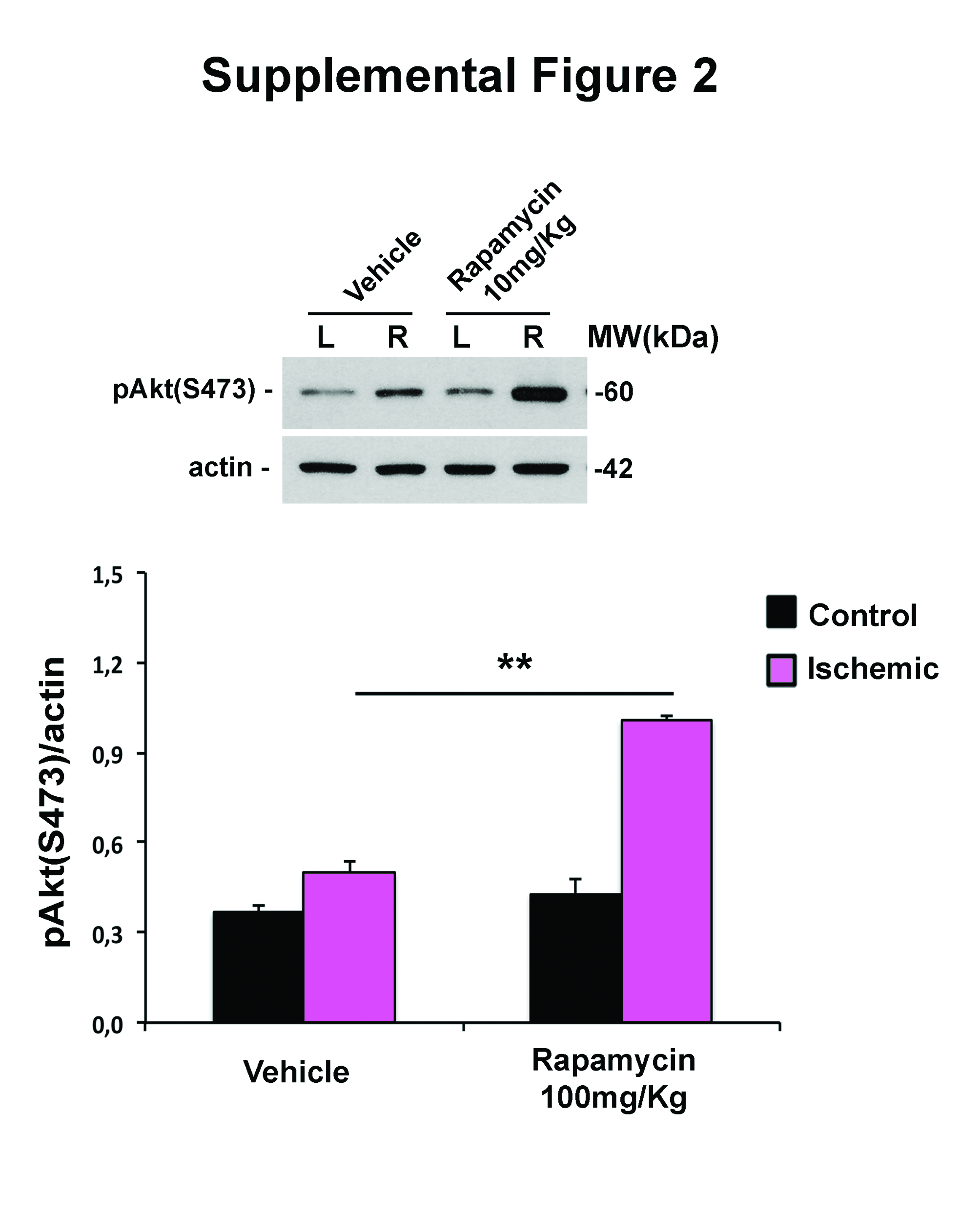

Supplement: Supplementary file 2 — Supplemental 2 [file 41419_2018_1044_MOESM2_ESM.tif]
