# Supplementary material for: Rapamycin and fasting sustain autophagy response activated by ischemia/reperfusion injury and promote retinal ganglion cell survival
Source: Cell Death Dis. 2018 Sep 24;9(10):981. doi: 10.1038/s41419-018-1044-5 (PMC6155349; doi:10.1038/s41419-018-1044-5)
Supplement: Supplementary file 3 — Supplementary materials [file 41419_2018_1044_MOESM3_ESM.docx]

**Supplemental Figure Legends**

**Supplemental Figure 1.** *Analysis of ATG proteins expression in retina of fasted mice.* Animals were fasted for 48 hours before the induction of retinal ischemia (I) and sacrificed at the end of the ischemic period. Contralateral non-ischemic retinas were used as control (C). (**A**) Representative immunoblotting showing the expression of Atg-related proteins in the retinas of mice fasted or subject to normal food intake (fed). Images from retinas of fed and fasted animals are from the same immunoblot that has been cut to remove irrelevant lanes. (**B**) ATG12/ATG5 conjugate was significantly upregulated in the control eye of fasted mice, while no significant changes were reported for beclin-1, ATG4 and ATG7 expression. Histograms represent the densitometric analysis of the bands normalized to loading control (GAPDH). Data are reported as mean ± s.e.m (n=3). P<0.05 (Student’s t-test).

**Supplemental Figure 2.** *Systemic treatment with rapamycin increases Akt phosphorylation in retina subjected to ischemia/reperfusion injury.* Rapamycin (10 mg/Kg) or vehicle were injected i.p. once a day for 6 consecutive days; ischemia was induced the fifth day in the right eye (I) and mice sacrificed after 24 hours. Contralateral retina was used as control (C). Western blot analysis shows the upregulation of Akt phosphorylation on Ser473 in the ischemic retina (I) from rapamycin-treated mice as compared to vehicle treated. Histograms report the results of densitometric analysis of the autoradiographic bands normalized on loading control (actin) from 3 independent experiments (mean ± s.e.m.). **P<0.01; (Student’s t-test). C, control non-ischemic retina; I, ischemic retina; MW: Molecular Weight.
